# Development of a Gait Independence Prediction Model in Patients With Stroke in a Convalescent Rehabilitation Ward: A Comparison of Decision Tree and Random Forest Models

**DOI:** 10.7759/cureus.105532

**Published:** 2026-03-19

**Authors:** Shogo Nakao, Tsuyoshi Motokawa, Takashi Nakamori

**Affiliations:** 1 Department of Rehabilitation, Okanami General Hospital, Mie, JPN; 2 Department of Rehabilitation Medicine, Toyota Memorial Hospital, Toyota, JPN; 3 Department of Occupational Therapy, Faculty of Health Sciences, Kansai University of Health Sciences, Osaka, JPN

**Keywords:** classification and regression tree, convalescent rehabilitation ward, gait independence, random forest, stroke

## Abstract

Aim: In this study, we aimed to examine the clinical utility of a classification and regression tree (CART) model for predicting independent ambulation at discharge based on physical function at admission to a convalescent rehabilitation ward, by comparing its performance with that of a random forest (RF) model. Seventy-three patients with stroke admitted to a convalescent rehabilitation ward were included.

Methods: Independent ambulation at discharge was defined using the Functional Independence Measure (FIM) locomotion item (walk/wheelchair): patients with a score ≥6 and ambulation as the primary mode of mobility were classified as independent, whereas those with a score <6 or wheelchair use as the primary mode of mobility were classified as nonindependent. The dataset was randomly divided into training (70%) and validation (30%) sets, and CART and RF models were developed using the training data and evaluated using the validation data.

Results: In the CART model, patients with a Trunk Impairment Scale (TIS) score <9 were classified as gait-independent when the FIM cognitive score was ≥30.5. Among patients with a TIS score ≥9, those aged <76.5 years were classified as independent, whereas those aged ≥76.5 years were classified as independent when the FIM cognitive score was ≥22.5. The area under the receiver operating characteristic curve was 0.832 and 0.856 for the CART and RF models, respectively, with no significant difference between the two models according to the DeLong test (p = 0.58).

Conclusion: These findings suggest that the CART model demonstrates discriminative ability comparable to that of the RF model and can hierarchically visualize the likelihood of gait independence based on admission assessments, thereby supporting clinical decision-making and intervention planning in convalescent rehabilitation wards.

## Introduction

Gait impairment is one of the most common functional deficits after stroke, affecting approximately 80% of patients with stroke [1]. Achieving independent walking is particularly important for stroke survivors in terms of independence, safety, and efficiency, as it contributes to quality of life and long-term health outcomes [2]. However, approximately one-quarter of patients with stroke fail to regain independent ambulation by three months after onset [3]. Therefore, in postacute inpatient rehabilitation wards (referred to as convalescent rehabilitation wards in Japan), accurately predicting the likelihood of gait independence at discharge from an early stage after admission is clinically meaningful, as it can inform goal setting, intervention prioritization, and discharge planning.

To date, logistic regression analysis has been widely used to predict independence at discharge [4]. Although logistic regression is a standard and robust analytical method, gait independence after stroke is influenced by multiple factors, including age, motor impairment, trunk function, and cognitive function [5,6], and interactions and threshold effects among these factors may exist. Given such complex relationships, conventional regression models may be insufficient to fully capture the mechanisms underlying functional recovery, highlighting the need to explore alternative analytical approaches [7].

In recent years, machine learning techniques have gained attention as methods to address these limitations [8]. Among them, decision tree analysis is characterized by its ability to present results in a tree structure, allowing for intuitive visual interpretation. Because selected explanatory variables are hierarchically arranged, relationships among factors can be clearly identified, facilitating clinical interpretation and practical application [9]. However, single decision tree models are known to be unstable, as their branching structures are highly dependent on the training data, which may lead to reduced predictive accuracy [10]. To overcome this instability, random forest (RF), an ensemble method that aggregates multiple decision trees, has been proposed [11]. Therefore, comparing decision tree analysis, which offers high interpretability, with RF, which is expected to provide superior predictive performance among machine learning methods, is meaningful for evaluating whether decision tree models achieve acceptable performance for practical clinical use.

The purpose of this study was to develop a decision tree model to predict gait independence at discharge based on physical function at admission to a convalescent rehabilitation ward and to examine the clinical utility of decision tree analysis by comparing its performance with that of a more accurate RF model.

## Materials and methods

Participants

Participants were 73 patients with stroke (age, 71.0 ± 17.5 years) selected from 249 patients who were admitted to the convalescent rehabilitation ward of our hospital in Japan between August 2023 and March 2025 and who did not meet the exclusion criteria. The exclusion criteria were as follows: 1) not independently ambulatory before admission, 2) already independently ambulatory at admission, 3) transfer to another hospital, 4) in-hospital death, and 5) missing data.

Data collection and measures

Baseline characteristics and physical function measures were retrospectively reviewed from electronic medical records. Baseline characteristics at admission to the convalescent rehabilitation ward included age, sex, stroke type (cerebral infarction or intracerebral hemorrhage), lesion side (right or left), and time since stroke onset. Physical function measures included the lower extremity motor score of the Fugl-Meyer Assessment (FMA) [12]. Trunk function was assessed using the Trunk Impairment Scale (TIS) [13], balance function was assessed using the Berg Balance Scale (BBS) [14], and cognitive function was assessed using the cognitive items of the functional independence measure (FIM) [15]. Mobility at discharge was evaluated using the FIM locomotion item (walk/wheelchair) [15]. Admission assessments were conducted within two days of admission, and discharge assessments were conducted within two days before discharge. All clinical evaluations were performed by physical therapists working in the convalescent rehabilitation ward.

Statistical analysis

Gait independence at discharge was defined using the locomotion item of the FIM. Patients were classified as gait-independent if they were able to walk independently, with or without walking aids, and had an FIM locomotion score of 6 or higher. Patients who required supervision or physical assistance for walking were classified as nonindependent, even if they were able to ambulate using walking aids. Patients whose primary means of mobility was wheelchair use were also classified as nonindependent.

To ensure a balanced distribution of the outcome variable (independent vs. nonindependent), the dataset was randomly divided into training (70%) and validation (30%) sets using the cvpartition function in MATLAB based on the outcome variable. The random seed was fixed at 2025 to ensure reproducibility.

A decision tree model was developed using classification and regression tree (CART) analysis with the training dataset, and the Gini index was used as the splitting criterion. To prevent overfitting, 10-fold cross-validation was applied to the training dataset, and the optimal tree complexity was determined by pruning [9]. For each candidate pruning level, 10-fold cross-validation was performed once within the training dataset, and the pruning level with the minimum cross-validation loss was selected. The tree-growth hyperparameters were MinLeafSize = 1, MinParentSize = 10, and MaxNumSplits = n − 1, where n denotes the training sample size. In addition, an RF model was constructed using 500 trees. The number of variables considered at each split (mtry) was set to the square root of the total number of explanatory variables (√p) [16,17]. Classification was performed based on predicted probabilities, with a threshold of 0.5. Based on previous studies indicating that gait independence after stroke is influenced by factors such as age, motor impairment, trunk function, and cognitive function [5,6], we selected explanatory variables reflecting demographic characteristics, stroke-related clinical factors, and physical and cognitive function. The explanatory variables included age at admission to the convalescent rehabilitation ward, sex, stroke type, lesion side, days since stroke onset, the lower extremity motor score of the FMA, TIS, BBS, and the cognitive items of the FIM. Data analysis was performed using MATLAB R2023b (MathWorks, Natick, MA). Model performance was evaluated in the validation dataset by calculating the area under the receiver operating characteristic curve (AUC), sensitivity, specificity, and their 95% confidence intervals for both models. AUCs for CART and RF were compared using DeLong’s test, which was performed in R (version 4.1.2; R Core Team, Vienna, Austria, 2021). The statistical significance level was set at 5%.

Ethical considerations

This retrospective observational study was approved by the Ethics Committee of Okanami General Hospital (approval number: 0001). The purpose and methods of the study were disclosed through institutional postings, and participants were provided with the opportunity to opt out of the study.

## Results

Participants

**Figure 1:**
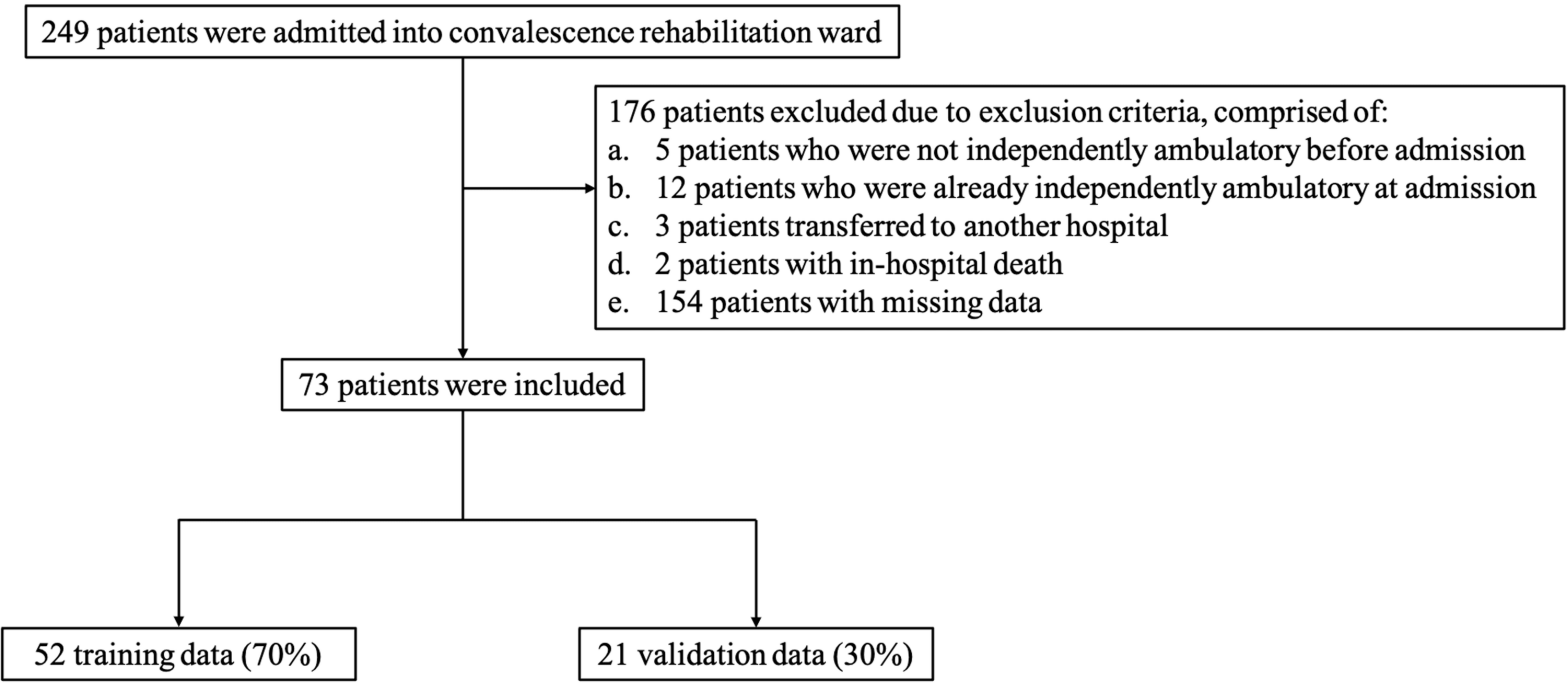
Participant flow diagram Of the 249 patients with stroke admitted to the convalescent rehabilitation ward, 176 are excluded according to the exclusion criteria, and 73 are included in the analysis. The included patients are randomly divided into a training set (n = 52) and a validation set (n = 21)

During the study period, 249 patients with stroke were admitted to the convalescent rehabilitation ward. Of these, 176 patients were excluded according to the exclusion criteria, and 73 patients were ultimately included in the analysis (Figure 1). The reasons for exclusion were as follows: five patients who were not independently ambulatory before admission, 12 patients who were already independently ambulatory at admission, three patients transferred to another hospital, two patients with in-hospital death, and 154 patients with missing data.

Baseline characteristics of the participants

Baseline characteristics of the overall cohort (n = 73) and the training (n = 52) and validation (n = 21) sets are summarized in Table 1.

**Table 1:** Baseline characteristics of the overall, training, and validation datasets Values are presented as median (interquartile range) or number (%) FMA: Fugl-Meyer assessment; TIS: Trunk Impairment Scale; BBS: Berg Balance Scale; FIM: Functional Independence Measure

Characteristic	Overall (n = 73)	Training (n = 52)	Validation (n = 21)
Age	76 (64-83)	76 (63-83)	76 (66-83)
Sex			
Female	39 (53.4%)	27 (51.9%)	12 (57.1%)
Male	34 (46.6%)	25 (48.1%)	9 (42.9%)
Stroke type			
Intracerebral hemorrhage	27 (37.0%)	23 (44.2%)	4 (19.0%)
Cerebral infarction	46 (63.0%)	29 (55.8%)	17 (81.0%)
Affected side			
Right	45 (61.6%)	31 (59.6%)	14 (66.7%)
Left	28 (38.4%)	21 (40.4%)	7 (33.3%)
Time since stroke onset	17 (13-23)	17 (14-24)	19 (11-22)
FMA	29 (23-32)	29 (16-33)	29 (25-31)
TIS	15 (9-19)	16 (8-20)	14 (10-17)
BBS	29 (6-42)	28 (6-44)	32 (9-40)
FIM cognition	23 (18-29)	24 (13-30)	23 (20-26)
Gait independence at discharge			
Independent	44 (60.3%)	31 (59.6%)	13 (61.9%)
Non-independent	29 (39.7%)	21 (40.4%)	8 (38.1%)

Structure of the CART model

**Figure 2:**
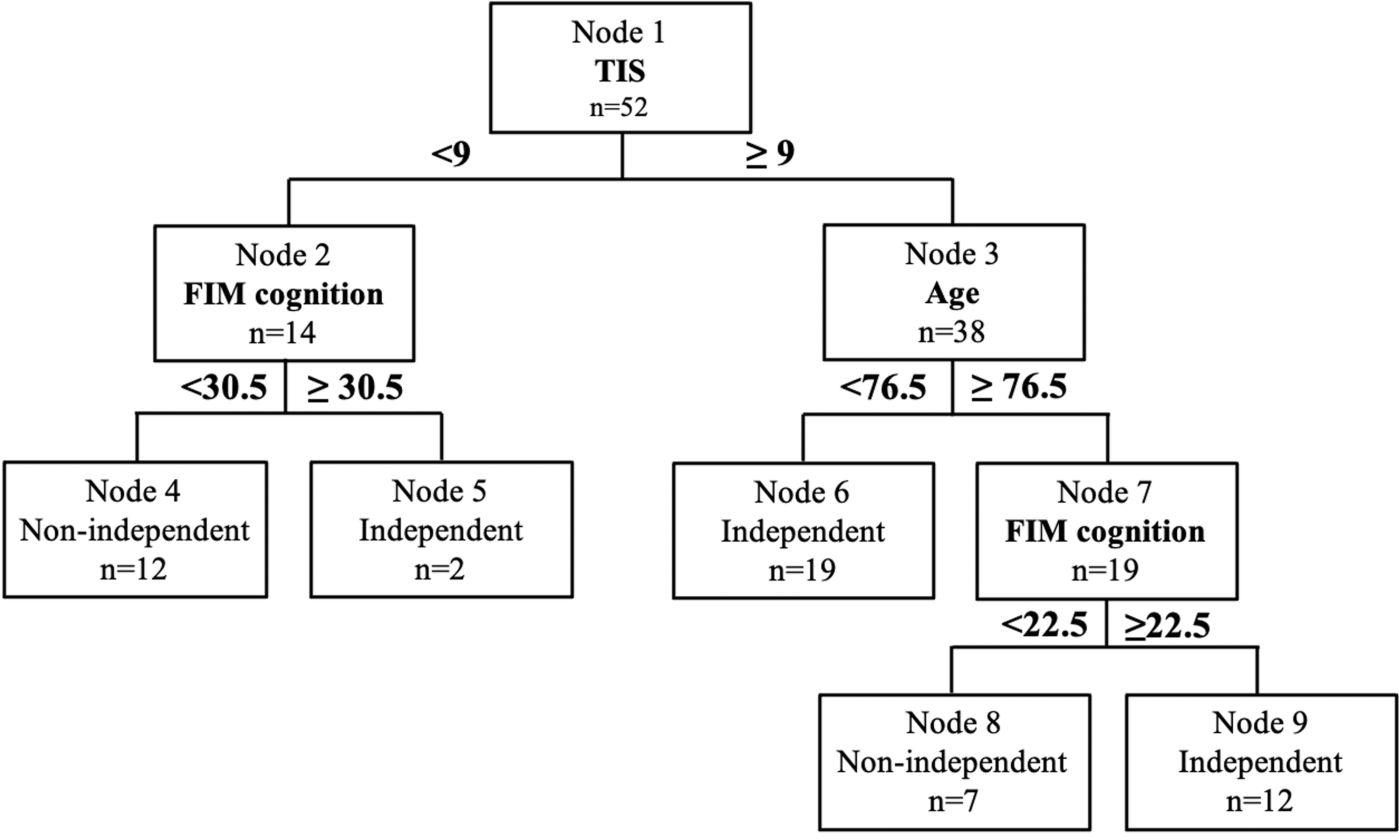
CART model for predicting walking independence at discharge The CART model constructed using the training dataset (n = 52) is shown. TIS is selected as the initial split variable, and patients with TIS <9 are further stratified by the FIM cognitive score. For patients with TIS ≥9, age is selected as the next split variable, and those aged ≥76.5 years are further classified according to the FIM cognitive score. Each node displays the number of patients and the predicted gait independence classification CART: classification and regression tree; RF: random forest; TIS: Trunk Impairment Scale; FIM: Functional Independence Measure

The structure of the CART model developed using the training dataset (n = 52) is shown in Figure 2. At the first split, the TIS was selected, dividing patients into those with TIS <9 and those with TIS ≥9. Among patients with TIS <9, the FIM cognitive score was selected as the next splitting variable. Patients with an FIM cognitive score <30.5 were classified as nonindependent (n = 12), whereas those with a score ≥30.5 were classified as independent (n = 2).

Among patients with TIS ≥9, age was selected as the next splitting variable. Patients aged <76.5 years were classified as independent (n = 19). For patients aged ≥76.5 years, the FIM cognitive score was further selected, with those scoring <22.5 classified as nonindependent (n = 7) and those scoring ≥22.5 classified as independent (n = 12).

Between CART and RF models

**Figure 3:**
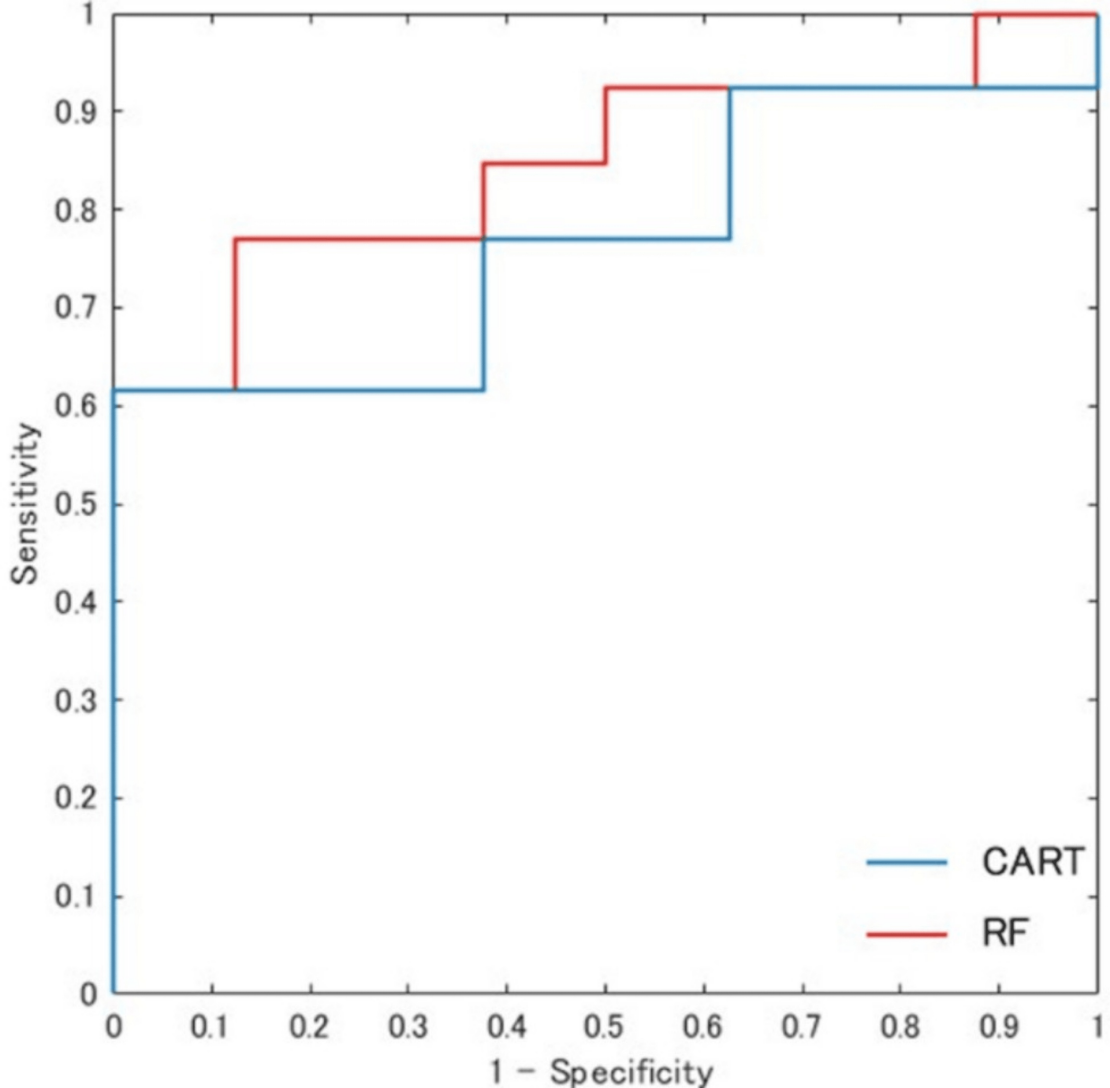
Receiver operating characteristic curves of CART and RF for predicting independent ambulation at discharge ROC curves generated using the validation dataset (n = 21) are shown. The AUC is 0.832 for the CART model and 0.856 for the RF model CART: classification and regression tree; RF: random forest; ROC: receiver operating characteristic; AUC: area under the curve

Receiver operating characteristic curves for the CART and RF models in the validation dataset (n = 21) are shown in Figure 3. The AUC was 0.832 for the CART model and 0.856 for the RF model. Comparison of AUCs using the DeLong test revealed no significant difference between the two models (p = 0.58) (Table 2).

**Table 2:** Comparison of AUC between CART and RF in the validation dataset CART: Classification and regression tree; RF: random forest; AUC: area under the receiver operating characteristic curve

Dataset	CART AUC	RF AUC	ΔAUC (RF - CART) (95% CI)	p value (DeLong)
Validation (n=21)	0.832	0.856	0.024 (-0.109, 0.061)	0.58

Sensitivity and specificity with 95% confidence intervals for both models are shown in Table 3. The CART model showed a sensitivity of 0.769 (95% CI, 0.462-0.950) and a specificity of 0.625 (95% CI, 0.245-0.915), whereas the RF model showed a sensitivity of 0.846 (95% CI, 0.546-0.981) and a specificity of 0.500 (95% CI, 0.157-0.843).

**Table 3:** Sensitivity and specificity of the CART and RF models in the validation dataset CART: Classification and regression tree; RF: random forest

Model	Sensitivity	Sensitivity_95CI	Specificity	Specificity_95CI
CART	0.769	0.462-0.950	0.625	0.245-0.915
RF	0.846	0.546-0.981	0.500	0.157-0.843

## Discussion

The present study aimed to examine the clinical utility of a CART model for predicting gait independence at discharge based on physical function at admission to a convalescent rehabilitation ward, by comparing its performance with that of an RF model. As a result, trunk function assessed by the TIS was selected as the first splitting variable in the CART model. Among patients with a TIS score <9, further stratification was performed based on the FIM cognitive score. In contrast, among patients with a TIS score ≥9, age was selected as the next splitting variable, and for those aged ≥76.5 years, the FIM cognitive score was additionally selected. These results visualized a hierarchical decision process associated with gait independence at discharge (Figure 2).

Furthermore, model performance in the validation dataset showed an AUC of 0.832 for the CART model and 0.856 for the RF model, with no significant difference between the two models according to the DeLong test (Figure 3, Table 2).

The selection of TIS as the top splitting variable in the CART model is consistent with previous studies reporting that trunk function is a key determinant of gait independence after stroke. TIS has been shown to predict gait independence in patients with acute stroke [5], and the present findings suggest that trunk function may also serve as an initial stratification factor at the time of admission to a convalescent rehabilitation ward.

Moreover, the selection of age as the subsequent splitting variable among patients with preserved trunk function (TIS ≥ 9), followed by cognitive function in older patients (≥ 76.5 years), indicates that even when trunk function is relatively preserved, aging and cognitive impairment may limit the acquisition of independent gait. The association of age and cognitive function with gait independence has been previously reported [5,18], and the hierarchical structure identified by the CART model in this study reflects the multifactorial nature of gait recovery after stroke.

In the comparison between CART and RF, the RF model demonstrated a slightly higher AUC than the CART model; however, no statistically significant difference was observed (Table 2). Recent studies have increasingly applied machine learning approaches such as RF to predict gait independence after stroke, in addition to conventional regression-based models [19]. From a clinical perspective, however, interpretability is as important as predictive performance. CART offers a clear advantage in this regard, as its decision rules can be presented as a tree structure, allowing clinicians to intuitively stratify patients based on admission assessments and directly link these findings to intervention planning and discharge support. The finding that CART achieved discriminative performance comparable to that of RF suggests that CART may serve as a practical decision-support tool in clinical settings. In addition, in the validation dataset, the RF model showed numerically higher sensitivity, whereas the CART model showed numerically higher specificity (Table 3). Because the confidence intervals were wide, these findings should be interpreted cautiously.

In this study, the CART model stratified gait independence at discharge by sequentially combining TIS at admission with age and cognitive function. These decision rules may provide clinically meaningful guidance for prioritizing interventions and identifying therapeutic targets early after admission to a convalescent rehabilitation ward.

Limitations

Several limitations of this study should be acknowledged. First, the validation dataset was relatively small (n = 21), which may have limited the statistical power to detect differences in predictive performance between the models. Second, this was a single-center, retrospective study, and several patients were excluded due to missing data. These missing data were primarily attributable to unperformed assessments or incomplete documentation rather than medical exclusion criteria. Therefore, the missingness may have been closer to missing at random. However, because this was a retrospective study, the exact missing-data mechanism could not be definitively determined, and other types of missingness cannot be ruled out. Under these circumstances, we conducted a complete-case analysis. Accordingly, the potential influence of selection bias on model development cannot be completely ruled out, and the results should be interpreted with caution. In addition, predictor assessments were performed by treating physical therapists during routine clinical practice and were therefore not blinded. Third, the length of stay was not considered in the present analysis. Because gait independence at discharge may be influenced not only by baseline physical function but also by the duration of inpatient rehabilitation, this factor should be considered when interpreting the results. Future studies should include external validation using multicenter datasets and larger sample sizes to confirm the reproducibility and generalizability of the CART model and its decision structure.

## Conclusions

In this study, we developed a CART model to predict gait independence at discharge based on physical function at admission to a convalescent rehabilitation ward and compared its performance with that of an RF model. In the CART model, trunk function assessed by the TIS was selected as the primary splitting variable, and the combination of age and cognitive function enabled further stratification of gait independence. The predictive performance of the CART model was comparable to that of the RF model, suggesting that CART offers a balance between interpretability and practical utility. CART may, therefore, represent a clinically valuable tool to support decision-making regarding intervention prioritization early after admission to a convalescent rehabilitation ward.
